# Letter to the editor in response to estimating the burden of antimicrobial resistance: a systematic literature review

**DOI:** 10.1186/s13756-018-0379-0

**Published:** 2018-07-31

**Authors:** Teresa M. Wozniak

**Affiliations:** Global and Tropical Health Division, Menzies School of Health Research and Charles Darwin University, Darwin, NT 0811 Australia

## Abstract

The systematic review published by Naylor et al. in April 2018 highlights methodological assumptions and biases that occur in studies investigating the burden of antimicrobial resistance (AMR). They note that, due to both the large diversity of statistical approaches and perspectives chosen, the current evidence base of the burden of AMR is highly variable. Certainly, these conclusions are valid and the authors present a very thorough analysis of the currently published literature with a broad array of drug-bug combinations. But readers are left with limited direction of estimating the current best available estimate of the health and economic burden of AMR. Such estimates are desperately needed to inform clinical management and for priority setting activities and initiative to curbing the global threat of AMR.

## Letter to the editor

It was with great interest that I read the recent systematic review by Naylor et al.(2018). This comprehensive systematic review investigated the approaches to estimating the burden of AMR for both gram negative and gram positive bacteria. They found 214 articles, comparing cases with resistant infections to both susceptible and uninfected controls.

The systematic review visualizes the eligible studies in a forest plots without performing a meta-analysis, which appears appropriate given the large heterogeneity of study design and outcomes measured. Despite this, authors go on to state that the majority of studies ‘..found resistance to be associated with higher mortality (Figures 2a and 2b).” Figure 2a depicts 24 comparison groups with only 11 studies directly measuring the impact of resistance on mortality, by comparing resistant cases with susceptible. From these direct comparisons, majority (*n* = 7; 63%) show odds ratio (OR) of mortality to cross the line of significant (OR < =1), compared to the other groups which show greater odds when compared to un-exposed controls. Hence, selections of comparator groups that will answer the research question best should be clearly stated and be consistent to ensure estimates remain comparable between studies.

Length of stay estimates depicted in Figure 4 of Naylor et al (2018), demonstrate the variety of methodologies used in studies (matching, multistate model, regression, significance test and stepwise calculation) generating excess length of stay (LOS) estimates ranging from as little as an additional 2.5 days [1] to a maximum of approximately 20 extra days in hospital [2]. These are not comparable without knowing whether this is determining the excess hospital stay of resistant cases compared susceptible controls or other control groups. Rigorous estimates of the length of stay are especially pertinent for determining the economic burden of infections on the healthcare system [3]. Timing of infection has by far the greatest independent effect on hospital costs compared to any patient-specific characteristics [4] [5]. In studies that have not fully adjusted for time-dependent bias, the magnitude of misclassification can inflate length of stay by as much as 9.8 days [4]. This would suggest that other than the included publications which fully adjusted for time of infection [6–8] or partially adjusted by matching based on time of infection [2], all other estimates presented in Figure 4 are generating longer LOS estimates leading to an inflated estimate of the cost burden of AMR (Table 3).

All studies investigating the burden of AMR should be assessed for risk of bias. We have developed a tool to prioritize the quality of studies based on previously identified methodological caveats when measuring the burden of AMR [9]. This tool assesses studies against three priority areas. The most important judgement of methodological quality of studies is how the study adjusted for LOS prior to infection onset and whether time-dependent bias was considered (Priority 1). This assessment needs to reflect whether the adjustment was made at the study-design phase (i.e. matching on LOS) or at the analysis phase (i.e. multistate modelling or adjusting for time to infection as a baseline covariate in regression analysis). Matched study designs are not considered to fully address time-dependent bias, as has been previously shown [4] . The second consideration is whether adjustment for comorbidities or severity of disease was made (Priority 2). Lastly, adjustment for inappropriate antibiotic therapy prior to infection (Priority 3) is considered to rank the quality of the studies.

In combination to the recommendations listed by Naylor et al. (2018) a prioritization of the quality of included studies would provide the reader with an appreciation of the strength of the estimates and allow a more informed judgement of the validity of AMR-attributable mortality (Figure 2), excess length of stay (Figure 4) and costs (Table 3). Such solutions should be in place to ensure the higher up the hierarchy the study design is positioned, the more rigorous the methodology and hence the more likely it is that the study design can minimize the effect of bias on the results. This would provide a level of clarity as to the ideal methodologies to use when designing studies and to guide the readers in the direction of deciding the best currently available estimates of burden of AMR.

## Abbreviations

AMR: Antimicrobial resistance
LOS: Length of stay
OR: Odds ratio
